# Simultaneous detection of downy mildew and powdery mildew pathogens on *Cucumis sativus* and other cucurbits using duplex-qPCR and HRM analysis

**DOI:** 10.1186/s13568-020-01071-x

**Published:** 2020-08-03

**Authors:** Kishore Babu Bandamaravuri, Ashish K. Nayak, Anu Sharma Bandamaravuri, Abdul Samad

**Affiliations:** 1 Department of Plant Pathology, Crop Protection Division, CSIR-Central Institute of Medicinal and Aromatic Plants, Lucknow, 226015 India; 2 Department of Microbial Technology, Crop Protection Division, CSIR-Central Institute of Medicinal and Aromatic Plants, Lucknow, 226015 India; 3 Microbial Genomics and Diagnostics Lab, Plant Pathology and Microbiology Division, Regional Plant Resource Centre, Bhubaneswar, Odisha 751015 India

**Keywords:** SYBR green, qPCR, Powdery mildew, Downy mildew, Cucumber

## Abstract

Powdery mildew and downy mildew are two devastating diseases on cucumber and other cucurbit crops caused by *Podosphaera xanthii* and *Pseudoperonospora cubensis*, respectively. Identification and detection of these pathogens from field and plant material could be significant for the selection of resistant varieties and formulation of disease management strategies. In the present study, a duplex qPCR assay developed for simultaneous detection and quantification of both pathogens from different samples. Two sets of species-specific primers developed for the detection of *P. xanthii* and *P. cubensis* pathogens by targeting the internal transcribed spacer (ITS) region of the rDNA gene cluster. The specificity of designed primers was also evaluated against the different microbial, plant, soil, and environmental samples. Initially, the individual assays for *P. cubensis* and *P. xanthii* were validated using their corresponding species-specific primers, which amplified the prominent and distinctive products of ~ 705 bp and ~ 290 bp size, respectively. SYBR green-based duplex real-time PCR assay was developed to detect and quantify both mildew pathogens from different field samples. The species-specific oligonucleotide primer sets showed high specificity with melt curve peaks at 85.83 °C and 88.05 °C, for *P. xanthii* and *P. cubensis*, respectively. The relative quantification and lowest detection limit of qPCR assays using tenfold diluted plasmid (*Csp*1 and *Csd*1) DNA were estimated (0.1 pg/µl) through a standard curve. In this study, the species-specific PCR and qPCR assays in both simplex and duplex formats have been validated successfully. These assays could be useful for efficient detection and quantification of mildew pathogens from the cucumber and other cucurbit crops.

## Key points

- The current study describes the identification and detection of two obligate biotrophic pathogens of cucurbit crops.
- The species-specific PCR assays provided a specific amplification of ~ 705 bp and ~ 290 bp products for *P. cubensis* and *P. xanthii*.
- The HRM analysis indicated that the primer sets were specific and simultaneously discriminated against *P. cubensis* and *P. xanthii* pathogens.
- The species-specific assays could be supportive diagnostic tools for plant pathologists, and plant breeders for detection and discrimination of both powdery mildew and downy mildew pathogens.

## Introduction

Cucurbits are one of the most cultivated vegetable crops worldwide, and a large number of annual species are under cultivation in tropical and sub-tropical regions (Judd et al. 2008). In India, the cucurbits are cultivated mainly as food crops, which share about 5.6% of the total vegetable production. *Cucumis sativus* (Cucumber) is one of the significant cucurbit crops under cultivation in India, and other cucurbit crops include gourds, melon, and pumpkin. Apart from food and vegetable use, many cucurbits seeds and fruits are reported to have medicinal properties due to the presence of secondary metabolite cucurbitacin (Kaushik et al. 2015). Notably, the fruits of *C. sativus* possess depurative, diuretic, and purgative properties and also used to treat blemished skin and heat rash (Bown 1995; Afari et al. 2012).

More than 200 known pathogens are infecting different cucurbit hosts (Zitter et al. 1996). The major fungal diseases are vascular wilt (*Erwinia tracheiphila*), gummy stem blight (*Mycosphaerella melonis*), anthracnose (*Colletotrichum orbiculare*), powdery mildew (*Podosphaera xanthii*), alternaria blight (*Alternaria cucumerina*), and downy mildew (*Pseudoperonospora cubensis*), (Watson and Napier 2009). Out of all fungal diseases, mildews are widespread and destructive plant diseases caused by obligate biotrophic pathogens, which produce large quantities of short-lived, asexual spores on the surface of host plant leaves. These asexual spores cause secondary infections and disperse through the air to infect fresh plants.

Downy mildew (DM), caused by *Pseudoperonospora cubensis* (Berk. et Curt.) Rostov., usually occurs in all cucurbit crops such as cucumber, bitter gourd, melons, pumpkin, and ridge gourd grown in open fields, net houses, and home gardens (Lebeda and Cohen 2011; Olczak-Woltman et al. 2011; Holmes et al. 2015). The severity and progress of the disease depend on favorable conditions like high humidity, temperature, light intensity, and source of inoculum. The preliminary symptoms appear on the upper surface of mature leaves as yellow angular spots and chlorotic lesions on the opposite side of the spot. Severely infected plants produce retarded/deformed fruits, which leads to a considerable loss of production. As the disease progress, the yellow spots became brown and then necrotic, which leads to leaf fall and death.

In the case of powdery mildew (PM) disease of cucurbit crops, two distinctly separate obligate pathogens such as *Golovinomyces orontii* (Castagne) V.P Heluta, and *Podosphaera xanthii* (Castagne) U. Braun and N. Shish. (Shishkoff 2000), causing significant economic losses in India and worldwide. Since both the pathogens produce similar symptoms and modes of infection, identification and differentiation of these pathogens become difficult. Though both are different species, morphologically distinct, and even epidemiological variations were reported, their molecular characterization and detection could provide an accurate estimate of each pathogen's distribution and economic impact (Nayak et al. 2019). In early stages, white circular powdery patches on either side of infected leaves and later whole leaf, petiole, stem, and branches of the plant were covered with white powdery spores. Several reports indicated that both mildew diseases were widespread worldwide, particularly in United States, China, Europe, India, and Israel, causing significant production loss in cucurbits (Colucci et al. 2006; Holmes et al. 2015; Lebeda and Urban 2004; Savory et al. 2011; Thomas 1996).

There are several established methods to control/manage both the mildew diseases such as application of chemical fungicides, the use of resistant or tolerant cultivars, and practicing crop rotation with a non-target host. However, the use of fungicides is not always feasible because of the high cost, and adverse effect on the environment. Moreover, excessive application of fungicides has shown development of resistance in several pathogens towards many chemical compounds (Waard et al. 1993; Hollomon and Wheeler 2002), which necessitates alternative or complementary methods that are effective against mildew pathogens and, reliable to crop ecosystem (Kiss 2003).

Early diagnosis of these infections could be a critical issue in order to implement effective strategies for controlling both mildew diseases (Wyenandt et al. 2015). Since both are caused by obligate pathogens of cucurbits, the possible way to detection of these pathogens could be either by typical disease symptoms or by DNA-based techniques. The conventional classification and identification of both these pathogens have been established (Goker et al. 2003; Waterhouse and Brothers 1981; Sitterly 1978) and molecular identification based on rDNA sequences has also been reported (Wang et al. 2008; Lee et al. 2016). However, due to the obligate nature of both pathogens, rapid and early detection of fungal pathogens in different plant materials and soil has an advantage over mere identification. The species-specific DNA-based molecular tools could be useful for early and accurate detection of plant pathogens (Gachon et al. 2004; Falacy et al. 2007). Therefore, PCR and quantitative real-time PCR (qPCR) based identification and detection of the unculturable fungal pathogens using species-specific oligonucleotides has become the most comprehensive, accurate, and rapid technology (Schenck et al. 2016; Lee et al. 2016). In recent advancements, qPCR has emerged with high resolution melting (HRM) analysis as an alternative to hydrolysis probe chemistry for detection and quantification of fungal pathogens (Summers et al. 2015; Lee et al. 2016; Zambounis et al. 2015). The duplex and multiplex PCR utilizes more than one set of primers within a single reaction; each primer set generates a specific size of amplicons, which segregate from other sets through electrophoresis (Chamberlain et al. 1988; Ioos et al. 2012; Bi et al. 2019).

In recent studies, though different specific primer sets of both *P. cubensis* and *P. xanthii* were designed by targeting the ITS region of rDNA (Wang et al. 2008; Lee et al. 2016; Pirondi et al. 2015), these assays were demonstrated separately for each pathogen. Further, the specificity and sensitivity of these assays against other fungi, commonly associated with cucurbit crops, were not determined. Therefore, a robust molecular detection assay to detect and distinguish both *P. cubensis* and *P. xanthii* simultaneously and accurately is of prerequisite. The current study focused on developing an SYBR green-based reliable and sensitive duplex qPCR assay for the identification and simultaneous detection of both pathogens from different samples.

## Materials and methods

### Pathogen collection and maintenance

In the present study, field surveys conducted during the winter season (Oct.–Feb.) in vegetable cultivating fields across different districts of Odisha, India in the year 2014–2015 and different locations nearby Lucknow, Uttar Pradesh, India in 2016–2017. Plant and leaf samples belonging to different cucurbit hosts infected by either powdery mildew or downy mildew diseases were collected (Table 1). The fungal and bacterial cultures used in the present study were also collected from different cropping and agro-forest areas (Table 2). The reference mildew obligate fungal pathogens (cannot be cultured on artificial media) such as *P. xanthii* CsKP07 and *P. cubensis* CsKD11 were established on respective hosts for propagation and maintained under glasshouse. The pathogens *P. xanthii* CsKP07 and *P. cubensis* CsKD11infected plant parts were collected for specimen preparation, and voucher specimens were stored at Microbial Germplasm Collection at Department of Plant Pathology, CSIR-CIMAP, Lucknow, India under the accessions MSCsPm180705 and MsCsDm180705 respectively. All other fungal and bacterial cultures used as representative test cultures were maintained on potato dextrose agar and nutrient agar slants until further use.

**Table 1:** Powdery mildew and downy mildew infected leaf samples of cucurbits hosts collected from different fields

Sl. no.	Isolate name and code	Host and place of collection	Accession no.^a^	Different PCR assays	
				PxK PCR	PcK PCR
1	*Podosphaera xanthii* (RPRCCs08)	*Cucumis sativus*, Puri	MN630275	+	−
2	*P. xanthii* (CmaKP15)	*Cucurbita maxima*, Lucknow, UP	NA	+	−
3	*P. xanthii* (Mc06)	*Momordica charantia*, Puri	NA	+	−
4	*P. xanthii* (Lc10)	*Luffa cylindrica*, Cuttack	NA	+	−
5	*P. xanthii* (LaKPm02)	*Luffa acutangula*, Keonjar	*KY319039*	+	−
6	*P. xanthii* (Ls01)	*Lagenaria siceraria*, Puri	*KU376473*	+	−
7	*P. xanthii* (CsKP07)	*C. sativus*, Keonjhar	MN630273	+	−
8	*P. xanthii* (CmKP04)	*Cucumis melo*, Lucknow, UP	NA	+	−
9	*P. xanthii* (LsKP02)	*L. siceraria*, Lucknow, UP	NA	+	−
10	*P. xanthii* (CsKP09)	*C. sativus*, Lucknow, UP	MN630271	+	−
11	*Pseudoperonospora cubensis* (CsKDM11)	*C. sativus*, Cuttack	MN630274	−	+
12	*P. cubensis* (RPRCCs04)	*C. sativus*, Keonjhar	*MH458898*	−	+
13	*P. cubensis* (LcKDm02)	*L. cylindrica*, Bhadrak	*KU041747*	−	+
14	*P. cubensis* (CmKD04)	*C. maxima*, Puri	NA	−	+
15	*P. cubensis* (Lc17)	*L. cylindrica*, Keonjhar	NA	−	+
16	*P. cubensis* (LaKDm03)	*L. acutangula*, Bhadrak	NA	−	+
17	*P. cubensis* (CsKD08)	*C. sativus*, Lucknow, UP	MN630272	−	+
18	*P. cubensis* (LA10)	*L. acutangula*, Sitapur, UP	NA	−	+
19	*P. cubensis* (CM04)	*C. maxima*, Lucknow, UP	NA	−	+

^a^Accession number in Italics are from other studies

**Table 2:** The genomic DNA of different microbial isolates used for validation of different species-specific PCR assays

Sl. no.	Isolate name	DNA/isolate code	Host and collection of place	Accession no.^a^
1	*Fusarium proliferatum*	Dr 04	*Dendrobium regium* orchid	*MF373328*
2	*Colletotrichum crassipes*	Pd13	*Pomatocalpa decipiens* orchid root	*MF373336*
3	*Penicillium* spp.	Ps05	*C. maxima*, Puri, Odisha, India	NA
4	*Colletotrichum siamense*	CIMAP:Up-72013	*Uraria picta*, CIMAP, UP, India	*KU925900*
5	*Curvularia pseudobrachyspora*	CIMAP: Ac-112017	*Acorus calamus*, CIMAP, UP, India	*MG645008*
6	*Rhizoctonia solani*	AG4	Plantago, CIMAP, UP, India	*KU253632*
7	*Pseudomonas fluorescens*	Pf B19	Soil, Puri, Odisha, India	NA
8	*Botryosphaeria ribis*	Em63	*Eria meghasinensis* orchid leaf	*MF373346*
9	*Fusarium oxysporum*	Fo02	*C. maxima*, Cuttack, Odisha, India	NA
10	*Diaporthe oncostoma*	Em71	*Eria meghasinensis* orchid stem	*MF373351*
11	*Alternaria alternata*	Aa04	*C. sativus*, Cuttack, Odisha, India	NA
12	*Fusarium solani*	Fs01	*C. maxima*, Cuttack, Odisha, India	NA
13	*Oidium heliotropii-indici*	Eh03	*Heliotropium indicum*, Odisha, India	NA
14	*Candida albicans*	Ca15	*C. maxima*, Puri, Odisha, India	NA
15	*Trichophyton terrespre*	Tt03	*C. maxima*, Puri, Odisha, India	NA
16	*Golovinomyces orontii*	Cg05	*Coccinia grandis*, Nayagarh,Odisha, India	*KY319040*
17	*G. orontii*	Cg02	*C. grandis*, Khordha,Odisha, India	*MG646282*
18	*Leveillula taurica*	Lt92	*Euphorbia heterophylla*, Odisha, India	NA
19	*Oidium heliotropii-indici*	Eh03	*Heliotropium indicum*	NA
20	*Oidium bonplandiani*	Ob93	*Croton bonplandianus*	NA
21	*Macrophomina phaseolina*	Mpk02	*Mentha arvensis*, Lucknow, UP, India	NA

^a^Accession number in Italics are from other studies

### Sample preparation and genomic DNA extraction

Powdery and downy mildew pathogen propagules such as conidia and sporangia, respectively were isolated from the corresponding infected living hosts maintained under glasshouse and purified for DNA extraction. Both conidia and sporangia collected into separate bottles containing 50 ml sterile distilled water (DW) at four °C with the help of vacuum suction (150 ppm). The samples were purified and subjected to genomic DNA extraction as per the protocol described by Nayak et al. (2019). In the case of test microbes used in this study (Table 2), genomic DNA extraction was carried out as described previously, and the DNA from the cultures reported in the previous study Nayak et al. (2019) were used as test samples for validation of PCR and qPCR assays.

### PCR amplification, cloning, and sequencing of rDNA gene cluster

The rDNA gene cluster, including ITS1, 5.8S and ITS2 regions of selected mildew pathogens listed in Table 1, were subjected to PCR amplification and direct sequencing using Big Dye™ Terminator v3.1 Cycle Sequencing kit (Applied Biosystems, USA), as described earlier (Nayak et al. 2018). The PCR products of both powdery and downy mildew pathogens such as *P. xanthii* CsKP07 and *P. cubensis* CsKD11 were then ligated into pGEM-T (Promega, USA) cloning vector according to manufacturer instructions. The clones/plasmids having insert of *P. xanthii* CsKP07 and *P. cubensis* CsKD11 were labeled as plasmid *Csp*1 and plasmid *Csd*1 respectively. The plasmid DNA of both pathogens were isolated and stored at − 20 °C for further use.

### Species-specific primers design and *in silico* analysis

Partial sequences of ITS 1, 5.8S, and ITS 2 regions of *P. xanthii* and *P. cubensis* along with other closely related fungal species of the genus *Podosphaera* and *Pseudoperonospora*, were retrieved from GenBank database. Multiple sequence alignment was performed separately for *P. xanthii* and *P. cubensis* pathogens using standalone software program MEGA6 (Tamura et al. 2013). The aligned sequences were visually checked for unique regions having conserved and specific to both *P. xanthii* and *P. cubensis*. These hallmark regions of both the alignments were subjected to Primer3 v4.1.0 online software (http://primer3.ut.ee) to design species-specific primers for both the pathogens (Untergasser et al. 2012). Thus two sets of candidate primers such as PxK F&R and PcK F&R were designed for both pathogens *P. xanthii* and *P. cubensis*, respectively (Table 3). All the primers were evaluated separately for theoretical specificity, and all the parameters such as G + C %, 3′-self complementarities, hairpin loop, and self dimerization analyzed and custom synthesized (IDT, USA). Besides, Primer-BLAST analysis (https://www.ncbi.nlm.nih.gov/tools/primer-blast/) was performed for all the designed primers using default parameters.

**Table 3:** Oligonucleotide primers designed for development of species-specific PCR and Duplex-qPCR assays.

Pathogen	Primers	Sequence (5ʹ– 3ʹ)	Length	Product size	Annealing temp. ( °C)	Melting curve peak ( °C)
*P. cubensis*	PcK F	GCTGGTTGATTACTGCTTGGCG	22	705 bp	58	88.05
	PcK R	CCGAAGCCACACAACACATAG	21			
*P. xanthii*	PxK F	CCCGTGTGAACTCTTATCTG	20	290 bp		85.83
	PxK R	GAGGGGTGTTCTGACGCTCG	20			

### Specificity and validation of species-specific primers

#### Conventional PCR assay optimization

The specificity of both the primer sets such as PcK F&R and PxK F&R of two mildew pathogens such as *P. cubensis* and *P. xanthii*, respectively, were validated through conventional PCR assays. Initially, the primer pairs' specificity was tested against the corresponding mildew isolates (Table 1). Subsequently, these assays were tested against non-mildew pathogens, includes *Alternaria* spp., *Aspergillus* spp., *Penicillium* spp., *Cladosporium* spp., *Botrytis* spp. etc. (Table 2). PCR was conducted in a 25 µl volume containing 12.5 µl of PCR master mix (Promega, USA), two µl of template DNA (~ 20 ng), 0.5 µl of each forward and reverse primers (10 µmol^−1^ each), and nuclease-free water to make up the volume. The PCR amplifications were conducted with an initial denaturation at 94 °C 7 min, followed by 35 cycles of 30 s at 94 °C, 30 s at 58 °C and 1 min at 72 °C and a final extension of 10 min at 72 °C. The DNA from healthy cucurbit plants and non-DNA template samples were used as negative controls. All the PCR products were analyzed on a 1.2% agarose gel along with a 1 kb DNA ladder (Thermo Fisher Scientific, India). All the experiments were repeated three times with three independent samples.

##### High-resolution melting (HRM) analysis and primer specificity evaluation

Real-time PCR validation of species-specific primers was performed using a 7500 Fast Dx real-time PCR instrument with SDS software (Applied Biosystems, USA). The reaction mix contained 12.5 µl of Maxima SYBR Green/ROX qPCR Master Mix (2×) (Thermo Scientific, USA) 400 nM of each primer, 20 ng of DNA template of each mildew isolate and nuclease-free water to reach a final volume of 25 µl. No template controls (NTC) were systematically included in triplicate. The thermal cycling conditions comprised an initial denaturation step of 7 min at 95 °C for 30 s and annealing at 60 °C for the 30 s. A melt curve analysis using a temperature gradient from 60 °C to 100 °C and a ramp speed of 0.5 °C/s and continuous fluorescent measurement was performed after the last cycle.

Species-specific qPCR assay performed, DNA isolated from pure cultures of both the mildew isolates CsKP07 and CsKD11, used as the positive control. Reactions containing DNA from healthy cucurbit plants and NTC reactions were used as negative controls. All qPCR assays were performed in triplicate. The expected size of both the mildew pathogens was confirmed by gel electrophoresis, as described above.

#### Optimization of specific PCR assays under duplex PCR mode

For the duplex PCR assay, the annealing temperature of both the designed primer sets was optimized through gradient PCR. The duplex PCR was conducted in a 25 µl volume containing 12.5 µl of Platinum^®^ Multiplex PCR master mix (2X)(Applied biosystems, USA), and reaction conditions were conducted as described in simplex PCR, except optimization of annealing temperature (58 °C), the concentration of the primer (2.5 pmol), and 0.5 µl template DNA. Duplex PCR products were analyzed on a 1.2% agarose gel with 1 kb ladder. All the experiments were repeated three times with three independent samples.

#### The sensitivity of qPCR assays and standard curve preparation

The sensitivity or limit of detection of species-specific primers under the duplex qPCR assay was determined by using the plasmid DNA obtained from *Csp*1 and *Csd*1 plasmids of both the mildew pathogens *P. xanthii* CsKP07 and *P. cubensis* CsKD11 respectively were prepared as mentioned earlier. The initial plasmid DNA concentration was adjusted to 100 ng/µl by using a NanoDrop™ 1000 spectrophotometer (Thermo Fisher Scientific, USA) and then serially diluted tenfold wise (1:10). The ten-fold serially diluted DNA was used as a target for the SYBR green-based qPCR, and thermal cycling conditions were followed as described above. A standard curve was drawn with linear regression between Ct and the log value of DNA concentration (Bandamaravuri et al. 2015). Appropriate negative controls were maintained; all the experiments were performed three times, and technical replicates maintained.

#### Detection of *P. cubensis* and *P. xanthii* from plant materials and soil samples

The species-specific duplex-PCR and qPCR assays were evaluated for the detection of targeted pathogens from plant and soil samples collected from different cucurbit fields (Table 4). Detection of both the pathogens was performed in both conventional PCR and qPCR assay under the duplex mode, as described above. Relative quantification of target pathogens was estimated through a standard curve derived between the linear regression of Ct and the log value of DNA concentrations. All these assays were analyzed in triplicate reaction.

**Table 4:** Detection of downy mildew and powdery mildew pathogens in different field and environmental samples

Sl. no.	DNA sample description	Duplex-PCR		Duplex-qPCR and HRM analysis	
		*P. xanthii*	*P. cubensis*	*P. xanthii* Conc. in ng/µl^a^/(85.83 °C)	*P. cubensis* Conc. in ng/µl^a^/ (88.05 °C)
1	Infected *C. sativus* leaf	+	+	1 /(√)	0.1/(√)
2	*C. maxima* leaf	+	−	0.15/(√)	−/(−)
3	*M. charantia* leaf, and stem	+	+	0.20/(√)	0.1/(√)
4	*L. cylindrica*, leaf	+	+	0.7/(√)	0.5/(√)
5	*L. acutangula*, leaf	−	+	−/(−)	0.5/(√)
6	*C. grandis*, leaf, and stem	−	−	− /(−)	−/(−)
7	*C. melo*, leaf	+	+	0.2/(√)	0.1/(√)
8	Surface soil from cucumber cultivating field	+	+	0.005/(√)	0.02/(√)
9	Seedlings of *C. sativus*	−	+	− /(−)	0.5/(√)
10	Infected *L. siceraria* dry, and fallen leaves	−	+	− /(−)	0.001/(√)

Duplex PCR indicating +-amplification, −-no amplification

^a^Relative quantification of target pathogens. (√)-Presence of signature peak and (−)-absence of peak in HRM analysis

## Results

### Specificity and validation of species-specific primers

**Fig. 1:**
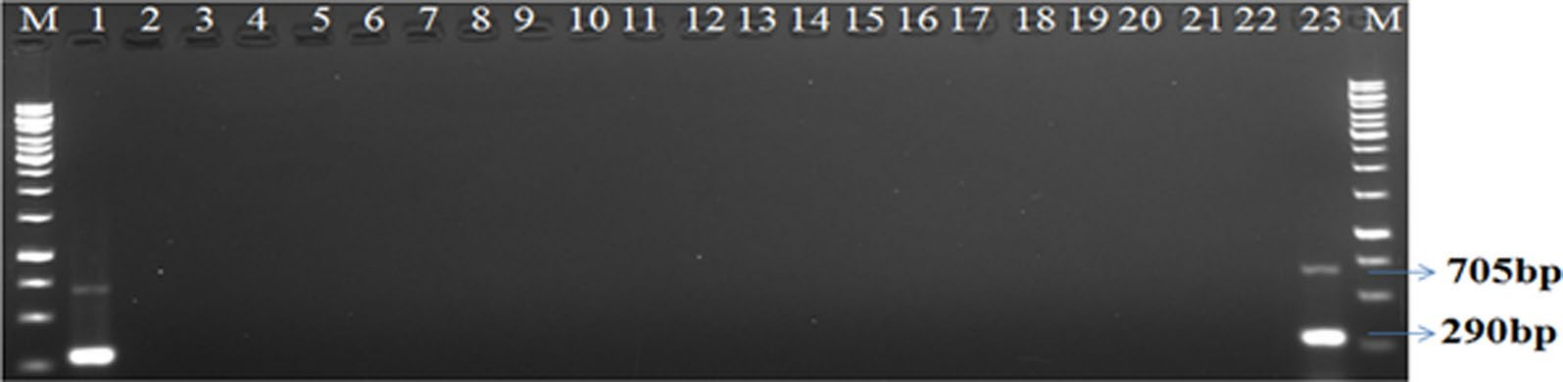
Validation of species-specific duplex PCR assay: Lane 1 and 23 indicate PCR amplified products of ~ 705 bp and ~ 290 bp from the genomic DNA of *P. cubensis* and *P. xanthii* isolates. Lane 2–22 represents test microbes listed in Table 2. M: 1 kb ladder

Initially, the specific primer sets such as PxK F&R and PcK F&R (Table 3) tested against their corresponding pathogens, and single monomorphic bands at ~ 290 bp and ~ 705 bp were obtained from *P. xanthii* and *P. cubensis*, respectively. Further, these species-specific primers or assays investigated against different test microbes obtained from different sources (Table 2). None of the test microbes showed amplification with the species-specific primers such as PxK F&R and PcK F&R (Fig. 1).

**Fig. 2:**
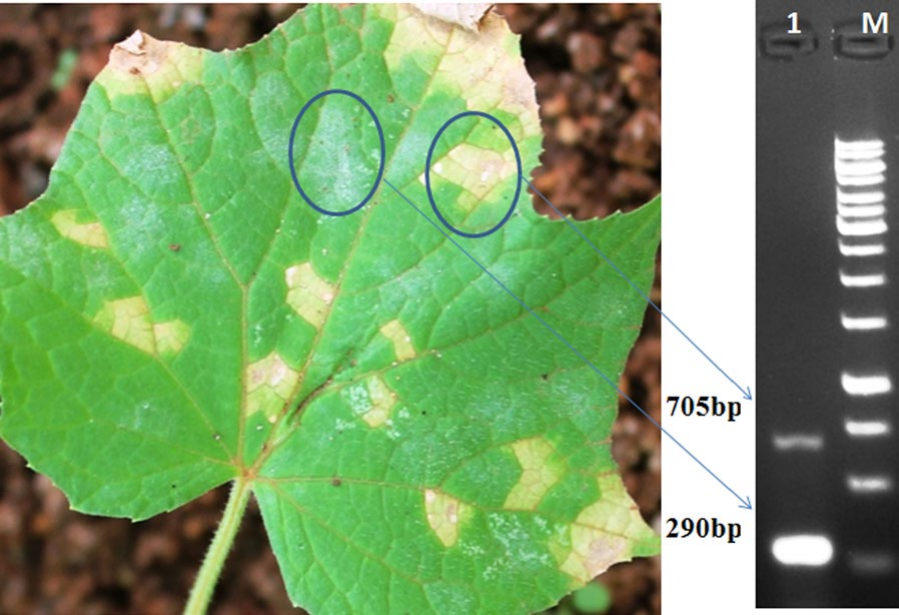
Illustration of species-specific duplex PCR assay: downy mildew and powdery mildew infection on *Cucumis sativus* leaf and gel picture (depicted from Fig. 1) indicating simultaneous detection of *P. cubensis* and *P. xanthii*

Both the species-specific PCR primer pairs (PcK F&R and PxK F&R) validated under individual PCR assays, as mentioned above, were subjected for validation under duplex PCR assay mode. The specificity of the duplex PCR assay was evaluated against different test microbial isolates as mentioned in Table 2, including the test microbes used in the Nayak et al. (2019) and genomic DNA obtained from dually infected cucumber (*P. cubensis* CsKD11 and *P. xanthii* CsKP07) leaf samples (Table 4) was used as a positive control. The duplex PCR assay showed amplification only in positive samples with two distinctly separated specific bands of ~ 705 bp and ~ 290 bp (Figs. 1, 2), and no amplification observed in test samples (Table 2).

### Real-time PCR HRM analysis

**Fig. 3:**
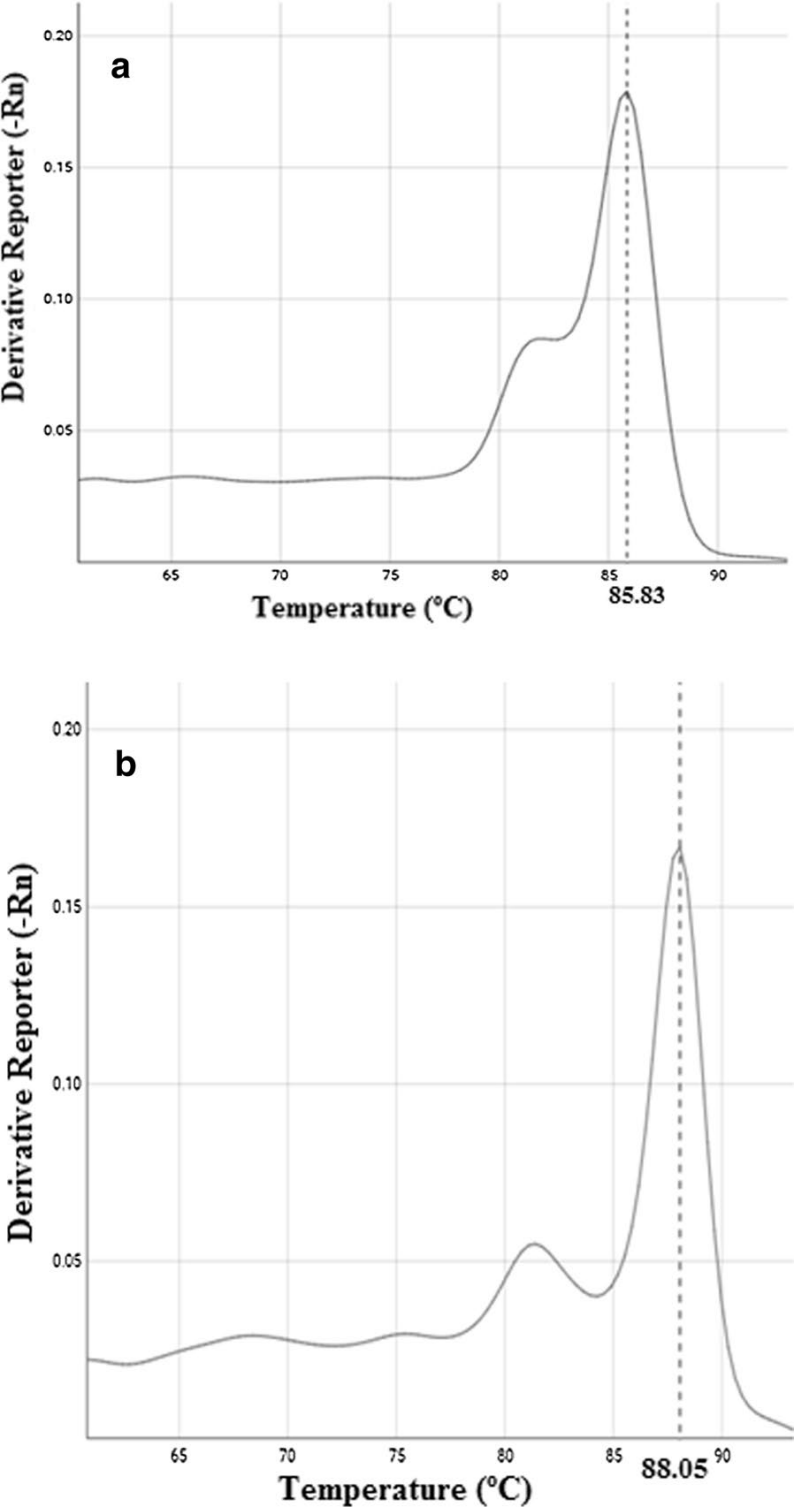
HRM analysis: SYBR green-based real-time PCR representing a single peak (**a**) at 85.836 °C for *P. xanthii*, and (**b**) at 88.05 °C for *P. cubensis *using species-specific primer sets PxK F&R and PcK F&R, respectively

The HRM analysis of both *P. cubensis* and *P. xanthii* was performed by using the genomic DNA of the respective pathogens (Table 1). The *P. cubensis* specific primers (PcK F&R) under SYBR green qPCR through HRM analysis produced a single peak at melting temperature (Tm) value of 88.05 °C (Fig. 3a). In the case of *P. xanthii*, specific primer (PxK F&R), the HRM analysis produced a single peak at a Tm value of 85.83 °C (Fig. 3b). In both assays, no amplification observed from the NTCs (no template control) and negative controls.

**Fig. 4:**
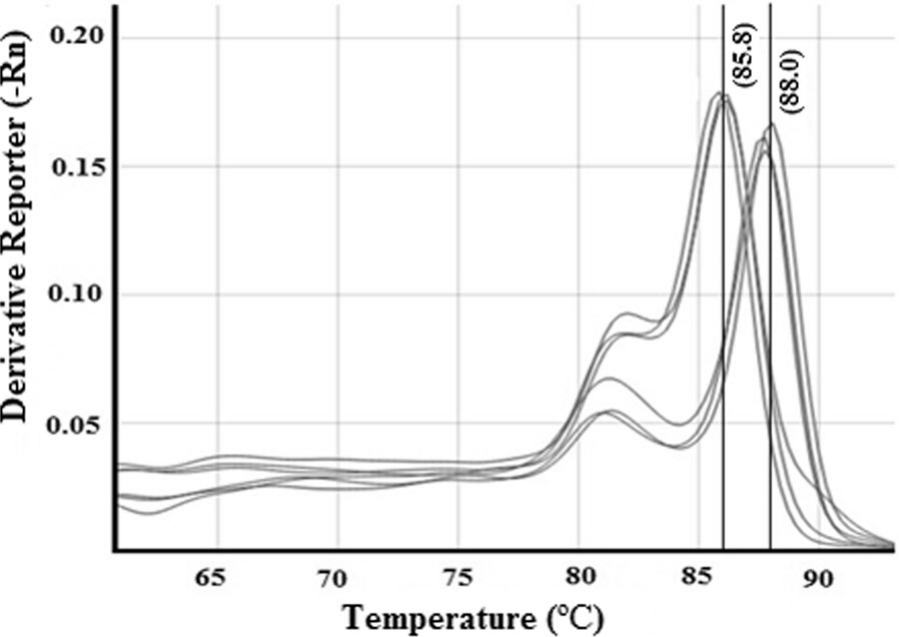
HRM analysis of duplex qPCR assay indicating two specific signature peaks at 85.8 °C for *P. xanthii* and 88.0 °C for *P. cubensis*

In the case of HRM analysis for a duplex qPCR assay, both the species-specific primers (PcK F&R and PxK F&R) and assay conditions were optimized for SYBR green qPCR format. The duplex HRM assay was performed using the genomic DNA of dually infected leaf samples (Table 4). The duplex HRM assay showed two separate melting peaks at Tm values of 85.8 °C, and 88.0 °C (Fig. 4), and the NTCs and negative control showed no amplification peaks.

The specificity of both simplex and duplex assays under HRM analysis determined by observing no fluorescent peak signals exceeding the baseline threshold and non-specific bands from different test microbes (Table 2). All the experiments performed in triplicate.

**Fig. 5:**
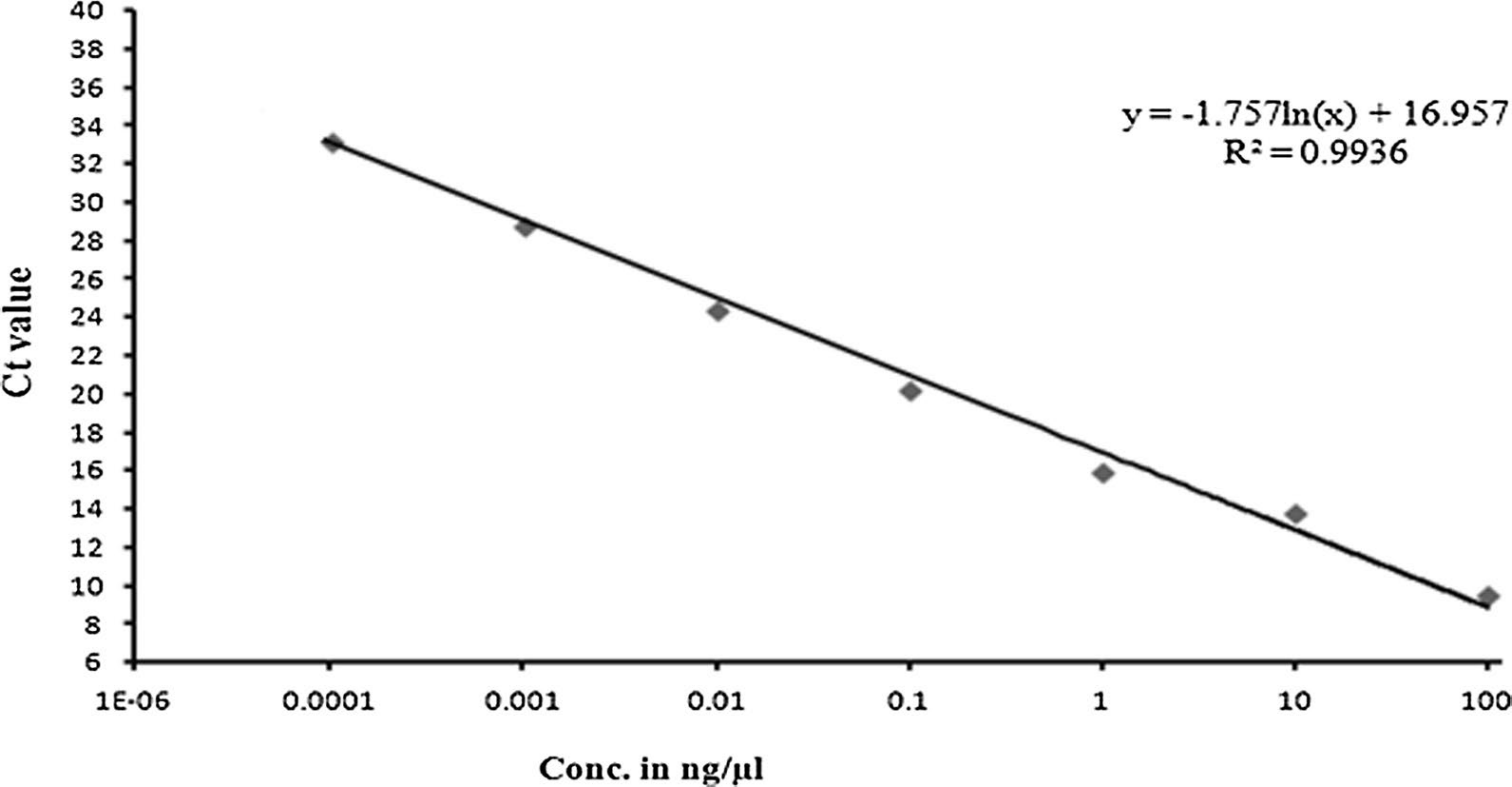
A standard curve indicating the limit of detection of *P. xanthii* species-specific qPCR assay obtained from 10-fold dilution of plasmid (*Csp*1) DNA

**Fig.6:**
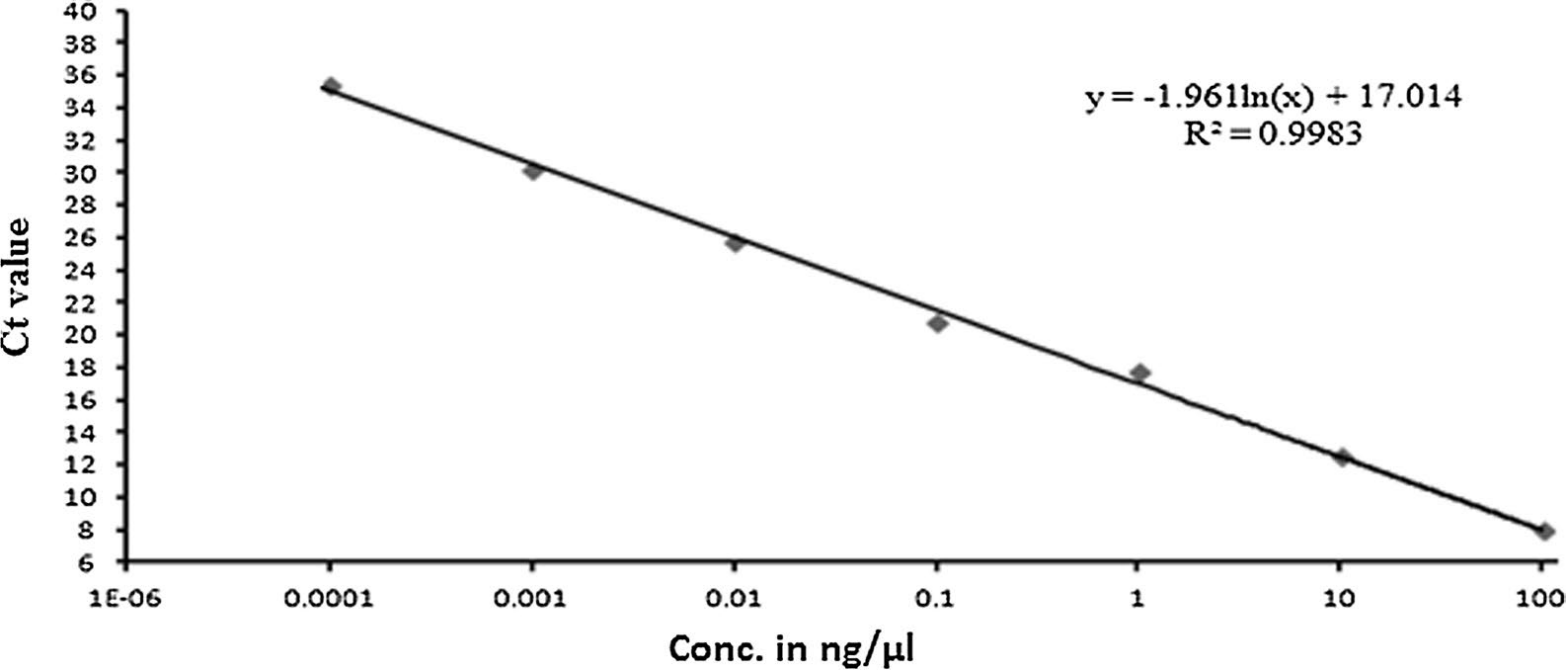
Limit of detection of *P. cubensis* specific qPCR assay, the standard curve derived from the 10-fold diluted plasmid (*Csd*1) DNA

Standard curves for both pathogens with the corresponding primer pair generated for evaluation of sensitivity or detection limit of each primer set under qPCR assay. Serial dilutions of the plasmid DNA of both pathogens provided a linear range of the standard curve between Ct value and the log of DNA concentration. The standard curve revealed that the PxK F&R primer set (*P. xanthii*) revealed a linear slope of ˗1.757 and a regression coefficient (R^2^) = 0.9936 with an amplification efficiency of 99% (Fig. 5). Similarly, in the case of *P. cubensis*, the standard curve between Ct and DNA concentration revealed a slope of ˗1.961 and R^2^ = 0.9983 and the amplification efficiency of PcK F&R primer set recorded as 99% (Fig. 6). The least detection of the target using the plasmid DNA of *Csp*1 (*P. xanthii*) and *Csd*1 (*P. cubensis*) estimated at 0.1 pg/µl, at Ct values around 34 and 35, respectively (Figs. 5, 6).

### Detection of *P. cubensis* and *P. xanthii* from the plant material and soil samples

**Fig.7:**
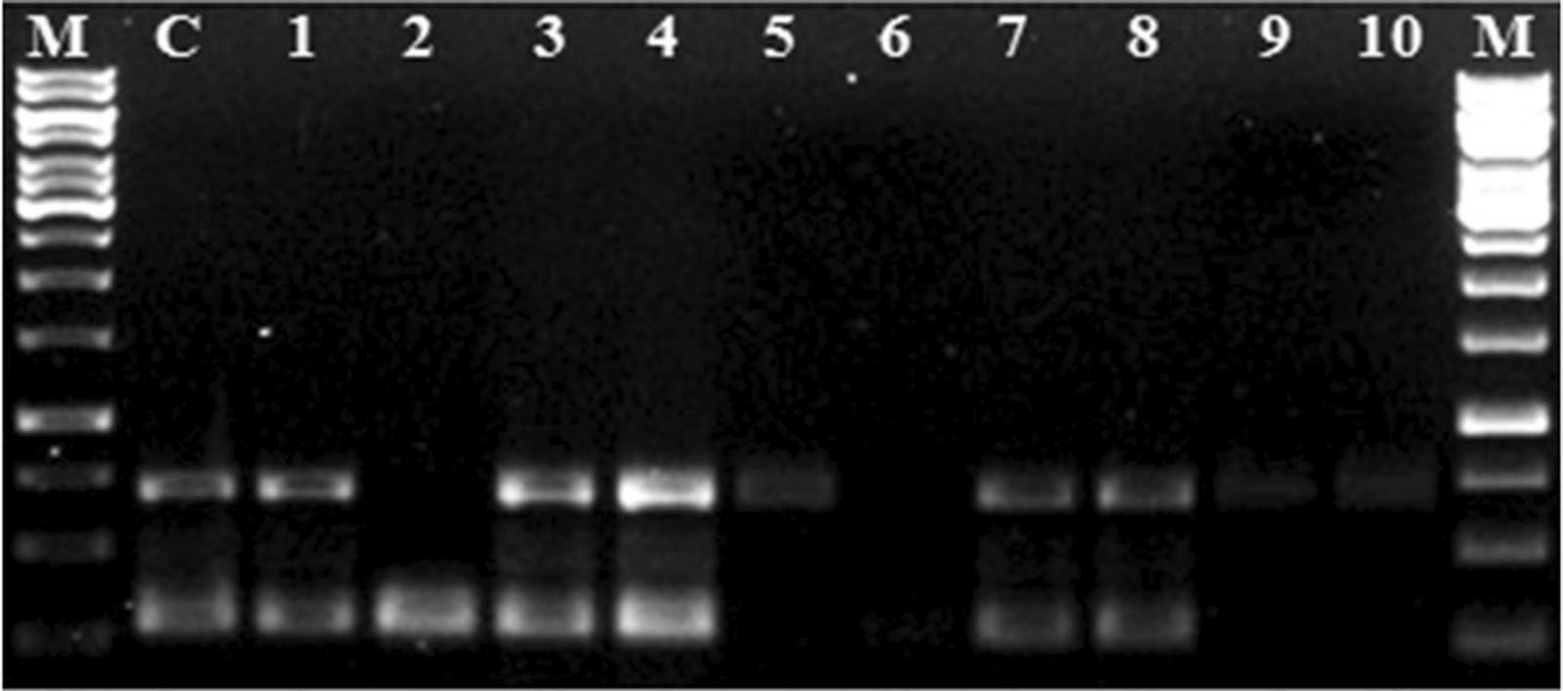
Evaluation of duplex PCR assay for field and environmental samples: Lane 1–10 represents different field samples listed in the Table 4. Lane C represents positive control for duplex PCR and M represents 1 kb ladder

Under optimized conditions, the SYBR green-based qPCR assay showed standard fluorescence amplification for both mildew pathogens in a separate assay representing exponential growth of PCR products, and standard curves obtained as described above. The HRM analysis of plant materials from *C. sativus*, *M. charantia*, *L. cylindrica*, *C. melo*, and surface soil samples from cucumber cultivating field showed two separate peaks at Tm value 85.8 °C and 88 °C, which represented the presence of dual infection of both pathogens such as *P. xanthii* and *P. cubensis*. While, the plant materials obtained from *C. maxima*, *L. acutangula*, *L. siceraria*, and seedlings of *C. sativus* showed a single peak as an indication of presence of a single pathogen. The relative quantification of the pathogens in each sample estimated through qPCR assay. The duplex PCR assay also provided similar results (Fig. 7), as indicated by HRM analysis. The plant materials of *C. grandis* showed no amplification in both PCR and qPCR assays (Table 4).

## Discussion

*Pseudoperonospora cubensis* and *P. xanthii* pathogens are devastating in nature with a broad host range of cucurbits and also challenging to identify by morphological, biochemical features, which are similar to many obligate pathogens (Lee et al. 2016). However, before taking any intervening control measures, it is vital to obtain an accurate picture of the phytosanitary situation (Wyenandt et al. 2015). Sometimes all cucurbit vegetables during the vegetative season can be easily confused with the disease symptoms; in some cases, both the mildew pathogens are infecting simultaneously on several cucurbits include gourds, melon, and pumpkin during favorable conditions and making it difficult to distinguish both the pathogens based on morphological symptoms particularly during early infection (Wallace et al. 2015). A rapid and reliable assay for detection and discrimination of *P. cubensis* and *P. xanthii* from field and plant material could be a prerequisite. Further, the powdery mildew disease caused by two different obligate fungal pathogens such as *G. orontii* and *P. xanthii* sharing common cucurbit hosts and showing similar disease symptoms, which made cucurbit powdery mildew disease more complex and challenging for disease-resistant varietal screening. To address this challenge, the current study developed a novel, sensitive and rapid duplex PCR assay and HRM analysis for simultaneous detection and quantification of *P. cubensis* and *P. xanthii* pathogens from different cucurbit crops (Table 1).

A reliable duplex PCR assay depends on the design and use of the primers specific to the intended target pathogen. In the current study, the species-specific primers for both the pathogens initially targeted on three prominent loci of the rDNA gene cluster, such as 28S, 18S, and ITS regions. Later the multiple sequence alignment of the ITS region of different species of Erysiphales and Peronosporales, closely related to both pathogens *P. xanthii* and *P. cubensis*, were found to have significant interspecies variations, which are suitable for the design of species-specific primers. The ITS region of the rDNA gene cluster has established as a suitable target for fungal species-specific primers due to their high copy number, sequence variability, and fidelity among pathogen species or subspecies. Several researchers reported that the identification of obligate fungi causing powdery mildew and downy mildew diseases by developing species-specific primers targeted on ITS region of rDNA (Wang et al. 2008; Pirondi et al. 2015; Bandamaravuri et al. 2015; Lee et al. 2016; Nayak et al. 2019).

In some cases, the sequence variations in the ITS region between closely related species are not always sufficient to define highly specific primers due to SNP variations (Choi et al. 2005; Lee et al. 2016). In recent studies, the transcriptome based species-specific genes targeted to enhance the specificity of the molecular detection assays (Rahman et al. 2019; Withers et al. 2016). In this study, two species-specific primer sets designed for both *P. cubensis* (PcK F&R) and *P. xanthii* (PxK F&R) targeting the ITS region of rDNA cluster. The specificity of both the primer sets was demonstrated by the specific amplification of ~ 705 bp and ~ 290 bp fragments from *P. cubensis* and *P. xanthii* using specific primers PcK F&R and PxK F&R, respectively. PCR amplification was not observed in non-target DNA samples and other closely related test microbes (Table 2), which indicated the specificity of both species-specific oligonucleotide primer sets.

Similarly, under the optimized parameters, the duplex PCR assay with PcK F&R and PxK F&R primer sets provided two distinctly separated PCR products of the expected size (Fig. 1). Both the specific primer sets were used separately in conventional PCR assays to evaluate their specificity. For estimation of detection limit and sensitivity of both the primer sets, validated through simplex and duplex PCR under SYBR green qPCR assay (Mackay et al. 2002). Further, the duplex qPCR assay successfully detected both pathogens *P. cubensis* and *P. xanthii* from different plant materials and soil samples obtained from cucurbit (Table 4). Thus the conventional and duplex PCR assay could be used to identify both pathogens from a complex microbial community other than plant-parasitic or obligate pathogens. The developed assays simultaneously detected and identified both pathogens directly from the DNA of leaf, stem, and field samples. The duplex PCR assay is suitable for detecting the fungal pathogen present in leaf or stem part of plants with or without symptoms (Figs. 2, 7). This assay could also be used to quantify the pathogen load and monitor the survival and spread of both pathogens at the early stage of disease development (Mercado et al. 2003; Rahman et al. 2017). This assay could get insights of disease epidemiology and of the disease, thereby used as a development of effective strategies for controlling diseases.

In recent studies, the HRM technique is being used for the rapid, accurate identification and discriminating between closely related pathogens in clinical biology and food microbiology fields of the study around the world (Kagkli et al. 2012). The advantages of this assay are higher sensitivity and better specificity, and the ability to quantify target pathogens from field samples. Compared with conventional PCR techniques, qPCR provides immediate results that are expressed with quantitative value, without the need for agarose gel electrophoresis. The HRM assay targeted on single-nucleotide variations at ITS and *cox*2 regions was developed to differentiate between two closely related cucurbit downy mildew pathogens *P. cubensis* and *P. humuli* (Summers et al. 2015; Lee et al. 2016). In the current study, though *P. humuli* was not used as a target in the detection assays, the other closely related and regionally important fungal pathogens were tested to evaluate the duplex qPCR assay specificity. The high resolution melting peaks in single and duplex HRM analysis stated about the primer specificity and also discriminate between *P. cubensis* and *P. xanthii* pathogens simultaneously. This assay also exhibited high efficiency and with the ability to identify as low as 0.1 pg/µl of the target DNA.

In conclusion, the optimized qPCR with HRM analysis is highly essential for detection or discrimination of both the target pathogens and also from other closely related pathogens even from a very fewer quantity of sample material. The species-specific primers designed and used in this study were specific and reliable for the detection and identification of both pathogens over simplex and duplex reaction setup. Also they provided a robust and rapid domino effect under both conventional and qPCR assay formats.

## Data Availability

The data supporting the results of this research work are included within the article. Data and materials including specimens and genetic material of obligate fungi, can be requested from the corresponding author.
